# Hot Melt Coating of Amorphous Carvedilol

**DOI:** 10.3390/pharmaceutics12060519

**Published:** 2020-06-06

**Authors:** Jacob Bannow, Lina Koren, Sharareh Salar-Behzadi, Korbinian Löbmann, Andreas Zimmer, Thomas Rades

**Affiliations:** 1 Department of Pharmacy, University of Copenhagen, Universitetsparken 2, DK-2100 Copenhagen, Denmark; jacob.bannow@sund.ku.dk (J.B.); korbinian.loebmann@sund.ku.dk (K.L.); 2 Institute of Pharmaceutical Sciences/Pharmaceutical Technology and Biopharmacy, Karl-Franzens-University of Graz, Universitätsplatz 1, A-8010 Graz, Austria; lina.koren@edu.uni-graz.at (L.K.); sharareh.salar@rcpe.at (S.S.-B.); andreas.zimmer@uni-graz.at (A.Z.); 3 Research Center Pharmaceutical Engineering GmbH, Inffeldgasse 13, A-8010 Graz, Austria

**Keywords:** hot melt coating (HMC), amorphous solids, solid-state, amorphous stability, amorphous drug delivery, dynamic in vitro lipolysis, carvedilol, tripalmitin

## Abstract

The use of amorphous drug delivery systems is an attractive approach to improve the bioavailability of low molecular weight drug candidates that suffer from poor aqueous solubility. However, the pharmaceutical performance of many neat amorphous drugs is compromised by their tendency for recrystallization during storage and lumping upon dissolution, which may be improved by the application of coatings on amorphous surfaces. In this study, hot melt coating (HMC) as a solvent-free coating method was utilized to coat amorphous carvedilol (CRV) particles with tripalmitin containing 10% (*w/w*) and 20% (*w/w*) of polysorbate 65 (PS65) in a fluid bed coater. Lipid coated amorphous particles were assessed in terms of their physical stability during storage and their drug release during dynamic in vitro lipolysis. The release of CRV during in vitro lipolysis was shown to be mainly dependent on the PS65 concentration in the coating layer, with a PS65 concentration of 20% (*w/w*) resulting in an immediate release profile. The physical stability of the amorphous CRV core, however, was negatively affected by the lipid coating, resulting in the recrystallization of CRV at the interface between the crystalline lipid layer and the amorphous drug core. Our study demonstrated the feasibility of lipid spray coating of amorphous CRV as a strategy to modify the drug release from amorphous systems but at the same time highlights the importance of surface-mediated processes for the physical stability of the amorphous form.

## 1. Introduction

With an increasing amount of lipophilic or hydrophobic low molecular weight drug candidates emerging from the pipelines of the pharmaceutical industry, pharmaceutical scientists are continuously confronted with the issue of poor aqueous drug solubility [1]. To enable a sufficient bioavailability of these drugs after oral administration, various formulation strategies have been developed [2,3], among which the conversion of the crystalline state of the drug into a high energy amorphous form has gained significant attention over the past decades [4,5,6]. Due to their high internal energy, most amorphous drug formulations exhibit the desired increase in solubility, which however is compromised by their tendency to recrystallize, often preventing their market access [7]. Consequently, the research focus was laid on the identification of factors that influence the physical stability of amorphous drugs. In this context, the mobility of molecules on amorphous surfaces has been found to play a major role in the physical stability of the amorphous form [8]. Due to their weaker or fewer intermolecular interactions at the solid-gas interphase and the resulting greater freedom of movement, molecules on the surface of amorphous solids are up to a million times more mobile than in the bulk [9]. Moreover, high molecular mobility on amorphous surfaces was found to correlate with the speed of surface crystal growth, resulting in fast recrystallization of the amorphous form [10].

To address the challenge of surface-induced recrystallization, different approaches have been investigated to slow down surface mobility of amorphous drugs [11], including the application of surface coatings. Wu et al. (2006) demonstrated that covering an amorphous indomethacin surface by glass slides significantly improved the physical stability of the amorphous form compared to samples with an air-exposed surface [10]. Different methods have since been established that mimic a more realistic coating process, for example, gold sputter coating [12,13,14] and layer-by-layer polyelectrolyte coating [14]. Despite the positive results of the above-mentioned techniques in preventing the crystallization of amorphous surfaces, the used materials were still lacking pharmaceutical relevance. To overcome this limitation, further studies utilized biopolymers which were deposited on amorphous surfaces by dip-coating from aqueous solutions based on electrostatic interactions, such as alginate on clofazimine [15], dextran sulfate on loratadine [16], gelatin on indomethacin and nifedipine [17], and chitosan on indomethacin [18]. The coating of amorphous materials with biopolymers not only resulted in improved physical stability but also was able to improve its powder flowability and dissolution performance [18]. In a recent study by Novakovic et al. (2020), the polymer coating approach from aqueous solutions was translated into a spray coating method using the pharmaceutically relevant polymer polyvinylpyrrolidone (PVP) for the coating of amorphous indomethacin compacts [19]. In contrast to the established use of PVP as an excipient for the preparation of amorphous solid dispersions, the direct targeting of the amorphous surface by coating with PVP reduces the overall polymer burden and enables a higher drug loading of the final dosage form. Moreover, the study demonstrated the feasibility of a scalable industrial process, like spray-coating, for the coating of amorphous solids and at the same time delaying the onset of recrystallization of the amorphous form.

While certain amorphous drugs tolerate temporary exposure to aqueous solvents (see above), others tend to recrystallize immediately after contact [20]. Consequently, the use of aqueous coating solutions cannot be considered a “one-fits-all” approach. Priemel et al. (2013) demonstrated that a simple dry physical mixture of amorphous indomethacin and polymer particles of an amino methacrylate copolymer (Eudragit E) could lead to the increased physical stability of the amorphous form, as long as the polymer could form a coherent coat on the amorphous surface [21]. Similar results were obtained by utilizing vibrational ball milling for the dry coating of the fast recrystallizing amorphous drug acetaminophen with carnauba wax [22]. Another solvent-free coating method that, in contrast to vibrational ball milling, enables a precise and scalable coating process, is hot melt coating (HMC) [23]. During HMC, the molten coating material is sprayed onto the substrate and solidifies on the substrate surface upon contact, forming a coherent coating layer [24]. Coating materials used in HMC applications are generally lipid-based excipients like waxes, hydrogenated vegetable oils, and their derivatives, e.g., fatty acids, partial glycerides, and polyoxylglycerides [23]. Lipid-based excipients are well suited for the HMC process due to their low melting points (<80 °C) and melt viscosities, compared for example, to synthetic polymers, which enable a continuous delivery of coating material by peristaltic pumps into the coating chamber [25]. Due to the hydrophobic character of HMC excipients, a prolonged-release from the coated material is achieved and can be modified towards an immediate release by the combination with surfactants or other more hydrophilic components [26]. Apart from the modification of release profiles, lipid coatings are widely used for taste masking [27], swallowability improvement, and moisture protection of oral formulations [28].

To our knowledge, no investigation of the HMC of amorphous solids and its impact on the physical stability and/or release from the coated amorphous material has been reported. In this study, we utilize a fluid bed hot-melt coating setup for the coating of amorphous carvedilol (CRV) with the aim to investigate the feasibility of lipid spray coating on amorphous surfaces and its impact on the physical stability and drug release during in vitro lipid digestion of the coated amorphous particles.

## 2. Materials and Methods

### 2.1. Materials

Carvedilol (CRV) was purchased from Cipla Ltd. (Mumbai, India) and Polysorbate 65 (Tween 65) was obtained from Croda Euro Ltd. (Fogras de la Selva, Spain). Tripalmitin (Dynasan 116) was kindly provided by IOI Oleo (Witten, Germany). Soy phospholipid (S-PC) was obtained from Lipoid (Ludwigshafen, Germany) and maleic acid, tris base, 4-bromobenzeneboronic acid (4-BBBA), bovine bile extract (B-3883) and porcine pancreatic lipase extract (P-1625) were purchased from Sigma–Aldrich (Saint Louis, MO, USA). Sodium chloride (NaCl), sodium hydroxide, potassium dihydrogen phosphate, and calcium chloride dihydrate were purchased from Merck (Darmstadt, Germany). Methanol (MeOH, HPLC grade) and acetonitrile (ACN, HPLC grade) were obtained from VWR (Herlev, Denmark).

### 2.2. Methods

#### 2.2.1. Preparation of Starting Materials

To obtain amorphous CRV as starting material for the HMC process, crystalline CRV was placed in aluminum pans and heated to 130 °C in a heating oven until a clear, crystal-free melt was formed. Subsequently, the pan was placed on an ice bath to facilitate the quench cooling of the molten CRV. The obtained amorphous CRV was equilibrated at ambient temperature and humidity for 30 min, followed by a manual grinding step using a mortar and pestle. The ground amorphous CRV was subsequently sieved into a particle size fraction ranging from 355–800 µm.

#### 2.2.2. Hot Melt Coating and Sample Storage

Hot melt coating of amorphous CRV particles in a fluid bed was performed at the RCPE in Graz using a Ventilus V-2.5 laboratory-scale system equipped with an Innojet IHD-1 hot melt device (Romaco Innojet GmbH, Steinen, Germany) (schematic provided in Supplementary Materials Figure S1). A batch size of 180 g of sieved amorphous CRV particles was used as core material for the coating trials. The following process parameters were used: 5 g/min spray rate, 1.1 bar atomizing air pressure, 45 m^3^/h airflow, 80 °C spray air temperature, and an inlet air temperature 25 ± 0.1 °C. Two different coating compositions were used: Tripalmitin containing 10% (*w/w*) PS65 (10% (*w/w*) PS65) and 20% (*w/w*) PS65 (20% (*w/w*) PS65). The theoretical coating mass ratio was set to 50% (*w/w*). Uncoated amorphous CRV particles and the coated products were stored at room temperature under dry (silica gel) and humid (75% rH, saturated NaCl solution) conditions. A third portion of the samples was vacuum-sealed and stored frozen at −20 °C to maintain fresh product conditions for the following in vitro evaluation. The obtained samples were shipped under controlled temperature and humidity conditions to Copenhagen for further analysis. The physical stability of coated and uncoated amorphous CRV particles was probed at regular intervals by X-ray powder diffraction (XRPD).

#### 2.2.3. Particle Size Analysis

The particle size of coated and uncoated amorphous CRV particles was analyzed using a QICPIC high-speed image analysis sensor (Sympatec GmbH, Clausthal-Zellerfeld, Germany) that was coupled to a RODOS/L dry disperser unit. A feeding rate of 30% with an injector diameter of 4 mm and an air pressure of 1 bar was used. Particle projections were captured at a frequency of 400 frames per second. The particle size was evaluated by calculating the diameter of a circle of equal projection area (EQPC).

#### 2.2.4. Differential Scanning Calorimetry

Differential scanning calorimetry (DSC) was carried out to study the thermal response of coated and uncoated amorphous CRV particles using a Discovery DSC (TA Instruments, New Castle, DE, USA). A sample mass of 3–5 mg was transferred to Tzero aluminum pans and sealed with pierced Tzero hermetic lids. All measurements were carried out using a heating rate of 10 °C/min from a starting temperature of −10 °C to an end temperature of 130 °C under a nitrogen gas flow of 50 mL/min. The obtained thermograms were analyzed with TRIOS software (TA Instruments, New Castle, DE, USA).

#### 2.2.5. X-ray Powder Diffraction

The physical stability of coated and uncoated amorphous CRV particles was analyzed by X-ray powder diffraction (XRPD) using a PANalytical X’Pert PRO X-ray diffractometer (PANalytical, Almelo, The Netherlands) with Cu Kα radiation (λ = 1.542 Å, current 40 mA, voltage 45 kV), operated in reflection mode. The samples were placed on aluminum sample holders and scanned from 5 to 35° (2θ) using a scan speed of 0.0673° s^−1^ and a step size of 0.0263° (2θ). Mercury 4.3.1 (CCDC, Cambridge, UK) software was used to calculate the theoretical diffractogram of the crystalline hydrate form of CRV, as no pure CRV hydrate crystals were obtained experimentally.

#### 2.2.6. Scanning Electron Microscopy and Energy-Dispersive X-ray Spectrometry

To study the morphology of coated and uncoated amorphous CRV particles, scanning electron microscopy (SEM) images were acquired using a Hitachi TM3030 tabletop microscope (Hitachi High-Technologies Europe GmbH, Krefeld, Germany) operated at an accelerating voltage of 15 kV. Samples were sputter-coated with gold (Cressington 108 auto, Cressington Scientific Instruments, Watford, UK) prior to SEM analysis.

The CRV distribution inside the stored coated amorphous CRV particles was studied by elemental mapping of cross-sections for the characteristic X-ray peak of nitrogen. The elemental distributions were investigated by environmental scanning electron microscopy (FEI Quanta 200 ESEM FEG, Hillsboro, OR, USA) combined with energy-dispersive X-ray (EDX) spectroscopy using an Oxford Instruments 80 mm^2^ X-Max silicon drift detector. The EDX images were analyzed using AZtecLive software (Oxford Instruments, Bristol, UK).

#### 2.2.7. Dynamic In Vitro Lipolysis

The release of CRV from coated and uncoated amorphous particles was studied using a dynamic in vitro lipolysis method that was previously described by Zangenberg et al. (2001) with minor modifications [29]. The amorphous particles were added to a temperature-controlled glass vessel (37 °C) resulting in a total CRV mass of 25 mg for each lipolysis run. A volume of 25 mL FaSSIF (tris-maleate buffer 2 mM, pH 6.5, bile salt 2.95 mM, phospholipids 0.26 mM, sodium chloride 50 mM) was added to the lipolysis vessel, which was equipped with an electrical stirrer. Lipid digestion was initiated after 1 min of predispersion by adding 5 mL of freshly prepared pancreatic extract (600 USP/mL) to the lipolysis vessel. To control the rate of lipolysis, calcium (0.5 M) was added at a continuous rate of 0.01 mL/min. The drop in pH, caused by the release of free fatty acids (FFA), was compensated by dosing a 0.4 M NaOH solution using a pH-stat apparatus (Metrohm Titrino 744, Tiamo Version 1.3, Herisau, Switzerland) to maintain a pH of 6.5 during the total run time of the experiment (120 min). Samples were withdrawn after 1, 5, 15, 30, 60, 90, and 120 min, and the lipase activity in the collected samples was immediately inhibited by the addition of 5 µl 4-BBBA (1 M, dissolved in MeOH). After centrifugation at 17,000 g for 15 min and appropriate dilution with ACN, the solubilized amount of CRV in the supernatant was quantified using HPLC.

#### 2.2.8. Coating Mass Ratio Determination

The determination of the coating mass ratio was carried out by HPLC analysis. Approximately 40 mg of the coated amorphous CRV particles were dissolved in 10 mL of MeOH. To ensure complete dissolution of CRV and disintegration of the coating shell, samples were ultrasonicated in a Branson 5510 ultrasonic bath (Branson Ultrasonics, Danbury, CT, USA) for 10 min and subsequently centrifuged at 17,000 g for 15 min. The concentration of CRV in the supernatant was quantified by HPLC after appropriate dilution with ACN.

#### 2.2.9. HPLC

The samples obtained during dynamic in vitro lipolysis and the determination of the core-to-shell mass ratio were analyzed using an Agilent 1260 Infinity chromatographic system (Agilent Technologies, Santa Clara, CA, USA) equipped with an ACE C18 column (Advanced Chromatography Technologies Ltd., Aberdeen, Scotland). The mobile phase consisted of acetonitrile and phosphate buffer (0.02 M, pH 2) (50:50% (*v/v*)) at a flow rate of 1.0 mL/min. The eluted CRV was detected at a wavelength of 242 nm.

## 3. Results and Discussion

### 3.1. Particle Characterization

**Figure 1:**
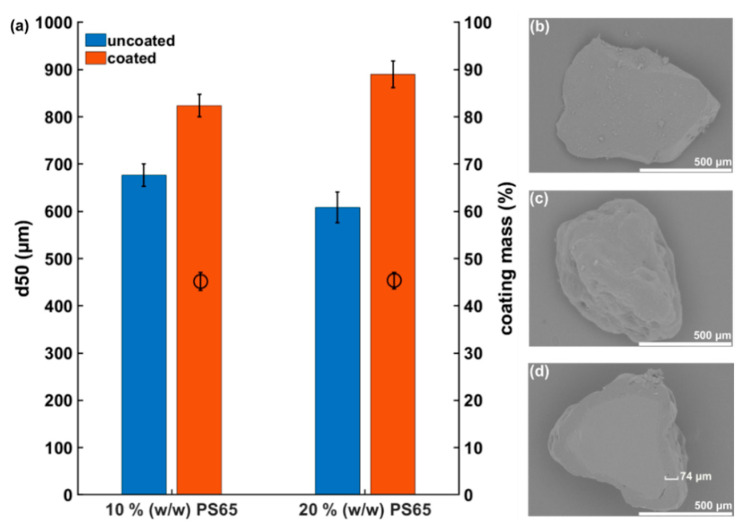
(**a**) Comparison of particle sizes of amorphous CRV starting material before and after hot melt coating and resulting coating mass ratios. Amorphous CRV particles were coated with 10% (*w/w*) PS65 and 20% (*w/w*) PS65, respectively. Results are presented as mean ± SD (*n* = 3). Representative SEM images of an uncoated amorphous CRV particle (**b**), a CRV particle coated with 10% (*w/w*) PS65 (**c**), and a CRV particle coated with 10% (*w/w*) PS65 as a cross-section including a representative determination of the coating layer thickness in (**d**).

Particles obtained after the manual grinding process and sieving of amorphous CRV showed an irregular particle shape with sharp edges (Figure 1b). The average particle size of the amorphous CRV starting material measured by high-speed image analysis was 676.2 ± 23.8 µm (10% (*w/w*) PS65) and 608.4 ± 33.0 µm (20% (*w/w*) PS65), respectively. In general, starting material particle sizes above 100 µm are desired for hot melt coating purposes to enable sufficient fluidization during the coating process [23]. The hot melt coating process with a theoretical coating mass of 50% (*w/w*) resulted in a particle size increase to 824.4 ± 23.9 µm (10% (*w/w*) PS65) and 890.3 ± 28.2 µm (20% (*w/w*) PS65), respectively (Figure 1a). As shown by the representative SEM images in Figure 1c,d, the formation of a coherent coating layer was achieved, despite the irregular shape of the amorphous CRV starting material. To further evaluate the resulting coating mass ratio, the CRV content of coated particles was quantified by HPLC. The obtained results were close to the theoretical value of 50% (*w/w*), with 45.2 ± 1.9% (10% (*w/w*) PS65) and 45.4 ± 1.7% (20% (*w/w*) PS65). The similar values obtained for both batches indicated a sufficient reproducibility of the coating process, leading to a coating mass ratio close to the targeted value. The data obtained from the coating mass ratio determination suggested a more homogenous coating result compared to the particle size analysis. This discrepancy can be explained by the irregular shape of the coated particles, leading to an under- or overestimation of the particle size increase. Ideally, particles should be as spherical as possible to aid the formation of a homogeneous coating layer [23].

### 3.2. Physical Stability Study

#### 3.2.1. Solid-State Characterization of Amorphous Particles Using XRPD

**Figure 2:**
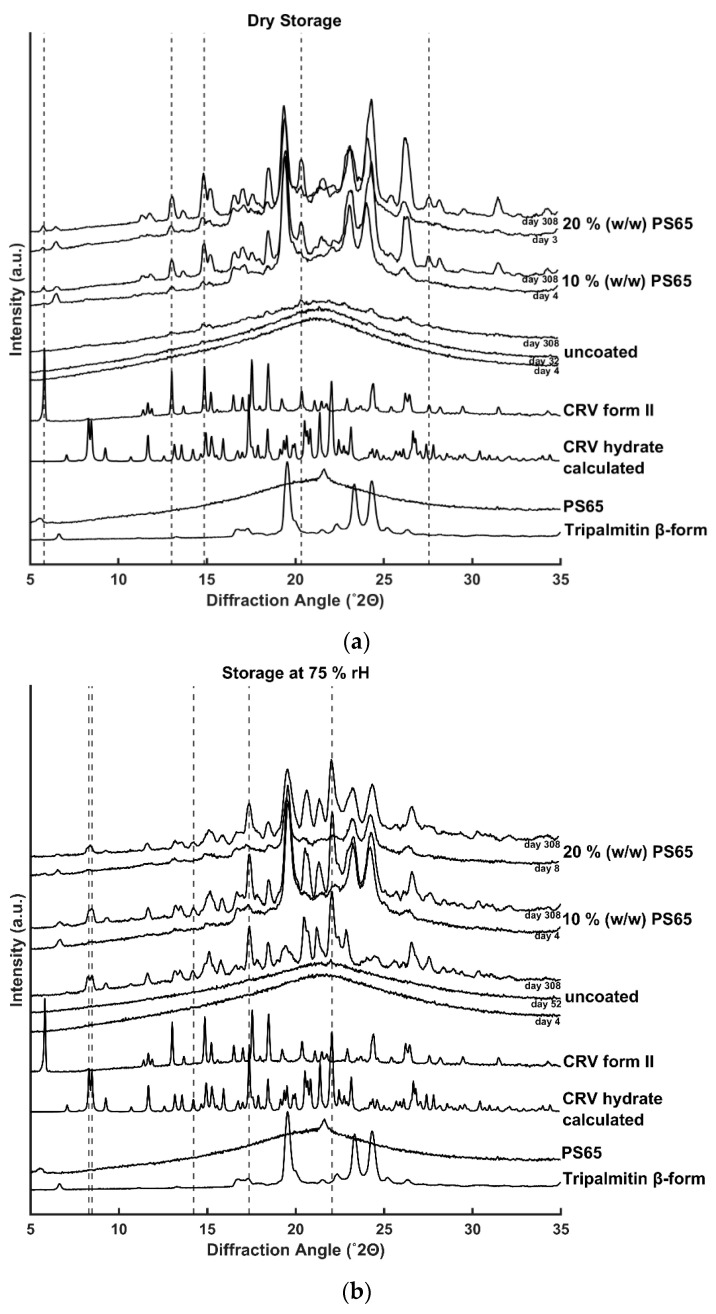
XRPD diffractograms of uncoated and coated amorphous CRV particles (10% (*w/w*) PS65) and (20% (*w/w*) PS65). The samples were stored at (**a**) dry condition and (**b**) 75% rH for more than 10 months. Characteristic diffractions of the polymorphic form II and the hydrate form of CRV are highlighted by the dashed vertical lines and serve as a guide for the eye.

The solid-state of the uncoated and coated amorphous CRV particles during storage was assessed by XRPD. As shown in Figure 2a,b, diffractograms of uncoated amorphous CRV starting material showed an amorphous halo, confirming the initial amorphous nature before the coating process. Uncoated amorphous CRV particles were physically stable at ambient temperature for 32 days at dry conditions and 52 days at 75% rH, respectively. Upon recrystallization, the samples stored at humid conditions showed characteristic Bragg peaks at 8.30, 8.48, 14.23, 17.36, and 22.05° (2θ), corresponding to the crystalline hydrate form of CRV, whereas storage under dry conditions resulted in recrystallization of the amorphous CRV into its anhydrous polymorphic form II with characteristic diffractions at 5.78, 13.01, 14.83, 20.35, and 27.55° (2θ) [30]. As shown in Figure 2b, unexpectedly, humid storage conditions delayed the onset of recrystallization of the uncoated amorphous CRV but resulted in a higher degree of crystallinity after 10 months of storage as shown by the higher diffraction intensities compared to the samples stored at dry condition, indicating different recrystallization kinetics of the different polymorphic forms.

In contrast to uncoated amorphous CRV, all coated particles showed diffractions corresponding to the anhydrous crystalline form II (dry storage) or the crystalline hydrate form (humid storage) of CRV already during the first measurements after the hot melt coating process, which were performed immediately after the sample shipment to Copenhagen (within 3–8 days after production, Figure 2). These results are indicating a decrease in the physical stability of the amorphous CRV compared to the uncoated samples. Due to the fast recrystallization of the coated particles, no conclusions about the impact of PS65 content or storage humidity on the physical stability of the coated particles could be drawn. However, it could be concluded that the applied lipid coating did not protect the amorphous CRV core from high humidity conditions. The crystalline hydrate form of CRV was only detected in samples that were stored at 75% rH, enabling the required water uptake for the observed hydrate formation. Coating formulations of tripalmitin and PS65 have previously been shown to be able to take up significant amounts of water due to the more hydrophilic character of PS65 (HLB 10.5) compared to the triglyceride tripalmitin [31]. To obtain effective protection of the coated particles from storage humidity, neat tripalmitin as the coating material would have to be used. However, this would lead to a strong retarding effect of the drug release, thus the amount of PS65 in the coating composition has to be carefully balanced between humidity protection and the desired release profile.

In addition to the detected crystalline CRV diffractions in coated particles, characteristic diffractions for the stable β-polymorph of tripalmitin at 19.4, 23.1, and 24.05° (2θ) were observed [32]. These findings indicate a fast conversion from the unstable α–polymorph (Supplementary Materials Figure S2) to the stable β-polymorph of tripalmitin after the coating process, which previously was found to be important for a reproducible release profile from the coated formulation during its shelf life [24].

#### 3.2.2. Investigation of the Chemical Composition of CrossSections Using SEM-EDX

**Figure 3:**
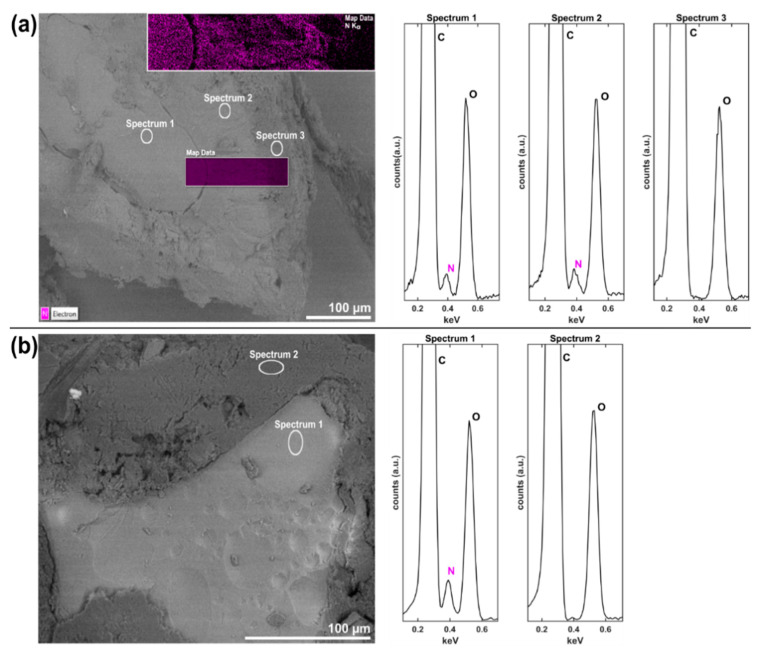
SEM cross-section images of coated amorphous CRV particles (20% (*w/w*) PS65) after 8 months of storage under (**a**) dry conditions at room temperature and (**b**) vacuum-sealed at −20 °C. The distribution of CRV is illustrated by EDX elemental mapping of nitrogen in a representative mapping window (purple window in (**a**)). The elemental composition at particle areas defined in (**a**) and (**b**) is shown in the EDX-spectra to the right of each panel.

To further investigate the detected recrystallization in coated amorphous CRV particles, SEM-EDX measurements of cross-sections were performed. Note that EDX analysis cannot provide direct information about the solid-state of the analyzed cross-sections but was rather utilized to visualize the distribution of CRV inside the coated amorphous particles, which was expected to provide valuable information about a potential mixing between the drug core and coating layer that might have occurred during the recrystallization process. As shown in the SEM image in Figure 3a, three distinct layers with contrast differences were observed, indicating differences in chemical composition. After 8 months of storage at dry conditions, the amorphous core of the coated particle was still clearly visible and showed no morphological indication for recrystallization emerging from the center of the particle. The lipid coating layer adjacent to the amorphous core separated into two different layers, which were not present in coated samples stored at −20 °C (Figure 3b), indicating recrystallization of the amorphous drug upon storage and not during the coating process itself. To verify the suspected recrystallization of amorphous CRV, resulting in a mixed layer comprised of crystalline CRV and the coating excipients, EDX analysis was performed. Due to the absence of nitrogen in the chemical structures of tripalmitin and PS65 and its presence in CRV (Supplementary Materials Figure S3), the detection of characteristic Kα radiation from nitrogen atoms was chosen as a marker for CRV. It is evident from spectra 1 and 2 in Figure 3a, that nitrogen was detected in the core and the mixed layer, but not in the outer coating layer. These results indicate a CRV crystal growth emerging from the surface of the amorphous core and growing toward the outer coating layer. The crystalline nature of the CRV detected in the mixed layer was confirmed by XRPD measurements (Section 3.2.1). Previous studies have demonstrated the ability of amorphous solids to grow crystals in an upward direction without penetrating the bulk, fed by a highly mobile layer of surface molecules [33]. This hypothesis is further supported by the surface area ratio between the amorphous core and the outer coating layers, as estimated from the SEM image in Figure 3a, indicating a reduced mass of the amorphous core due to the crystal growth into the surrounding lipid layer. Additionally, EDX mapping was performed on a representative measurement window covering all three layers. As previously suggested by the local spectra (Figure 3a), and in contrast to the outer layer, nitrogen was detected in the amorphous core and mixed layer, as visualized by the purple coloring in Figure 3a.

### 3.3. Probing the Thermal Properties of Amorphous Particles by DSC

**Figure 4:**
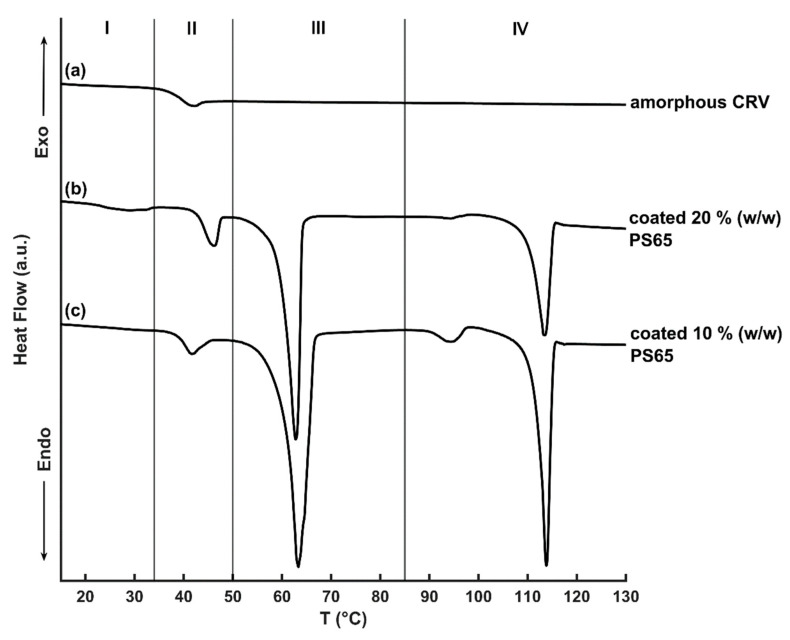
DSC thermograms of freshly amorphous CRV (**a**) and coated amorphous CRV particles (20% (*w/w*) PS65 (**b**) and 10% (*w/w*) PS65 (**c**)) after 1 month of storage under dry conditions. The Zones I–IV correspond to the following thermal events: I—melting of PS65, II—glass transition of amorphous CRV, III—melting of tripalmitin and IV—recrystallization and/or melting of crystalline CRV.

The thermal analysis of neat amorphous CRV showed a glass transition at 38.6 °C, which was in accordance with previously published results [34]. During further heating, no recrystallization or melting event was observed (Figure 4a). For coated amorphous CRV particles (20% (*w/w*) PS65) a weak and broad melting endotherm with the onset at 22.1 °C was measured, corresponding to the melting temperature (T_m_) of PS65 [31] that was not detected for particles coated with 10% (*w/w*) PS65, indicating the limit of detection of the employed method (Figure 4b,c). Upon further heating, the glass transition of the amorphous CRV core was detected, which was slightly increased compared to uncoated amorphous CRV (40.0 °C for 10% (*w/w*) PS65 and 44.0 °C for 20% (*w/w*) PS65) and followed by a weak endothermic event owing to the structural relaxation of the amorphous form during storage [35]. The melting onset of the coating’s main component, tripalmitin occurred at 59.7 °C (10% (*w/w*) PS65) and 60.4 °C (20% (*w/w*) PS65), respectively, corresponding to the thermodynamically stable β-polymorph of tripalmitin [24]. The detection of the β-polymorph in coated amorphous CRV samples was in agreement with the XRPD measurements described in Section 3.2.1. For coated amorphous particles (10% (*w/w*) PS65) a small melting endotherm at approx. 91 °C was detected, which corresponds to the melting of the CRV hydrate form (Supplementary Materials Table S1). The detection of CRV hydrate in the dry-stored coated samples was unexpected, but suggested a mixed crystallization behavior of amorphous CRV into both polymorphic forms, with the quantity of the formed crystalline hydrate form staying below the limit of detection of the XRPD measurements in Section 3.2.1. In contrast to the uncoated amorphous CRV, a broad recrystallization exotherm for coated particles with an onset temperature of approx. 94 °C was observed, followed by a melting event with an onset at 110.3 °C (10% (*w/w*) PS65) and 111.9 °C (20% (*w/w*) PS65), respectively, corresponding to the T_m_ of the polymorphic form II of CRV. The observed values were slightly lower than those measured for the pure crystalline CRV II (Supplementary Materials Figure S4) and the previously reported value of 115 °C by Prado et al. (2014) [30]. These deviations could be explained by the overlapping recrystallization and melting events, making a precise determination of the T_m_ onset more difficult. Moreover, the melting enthalpy of crystalline CRV was larger compared to the preceding recrystallization enthalpy, indicating the presence of crystalline CRV in coated particles at the time of DSC analysis, as also previously detected by XRPD in Section 3.2.1. It can be assumed that the crystalline drug content acted as a seed for recrystallization upon heating of the amorphous core that was not evident in uncoated amorphous CRV. However, no conclusions about the ability of the crystalline lipid coating alone to initiate recrystallization of the amorphous fraction could be made, due to the presence of crystalline CRV at the point of the first DSC measurement. Additional DSC studies, investigating the thermal behavior of neat amorphous CRV in combination with the coating excipients, could provide valuable information about possible interactions between the coating material and amorphous CRV that led to the recrystallization of the amorphous drug during storage, as observed in the physical stability study in Section 3.2.

### 3.4. Investigation of Lipid Coating Digestion by Dynamic In Vitro Lipolysis

**Figure 5:**
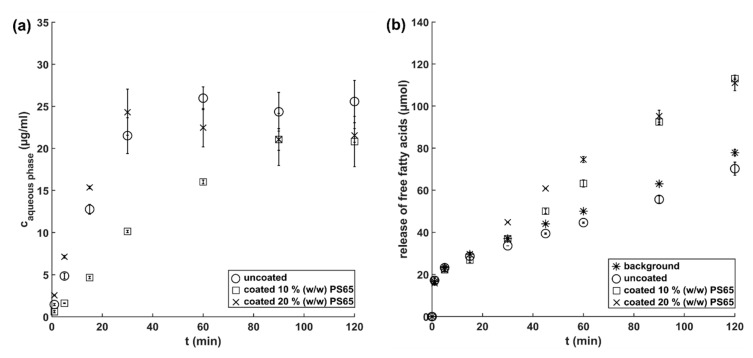
(**a**) Concentration of CRV in the aqueous phase of the lipolysis medium and (**b**) release of FFA during dynamic in vitro lipolysis of uncoated amorphous CRV particles (circle) and coated amorphous CRV particles (10% (*w/w*) PS65 (square) and 20% (*w/w*) PS65 (cross)). The titration of FFA released during dynamic in vitro lipolysis of lipids present in blank FaSSIF is shown by the asterisk. Results are presented as mean ± SD (*n* = 3).

To study the release of CRV from uncoated and coated amorphous CRV particles in a biorelevant setting, dynamic in vitro lipolysis was performed. It has previously been shown that the drug release from solid lipid formulations in a lipolytic set-up can differ significantly from conventional dissolution testing in simplified buffer media, due to the digestion of the lipid matrix by the pancreatic lipase [36]. To monitor the suspected hydrolysis of the ester bonds of tripalmitin and PS65 by the pancreatic lipase, the release of FFA was measured throughout the experiment (Figure 5b).

Coated amorphous CRV particles were stored at –20 °C prior to the in vitro lipolysis measurements to ensure the physical stability of the amorphous core and comparability with uncoated amorphous samples. Coated amorphous CRV particles (20% (*w/w*) PS65) showed a similar fast drug release compared to uncoated amorphous particles, reaching their maximum concentrations after approximately 30 min of digestion. In contrast, coated amorphous particles (10% (*w/w*) PS65) showed a significantly slower release, leading to comparable CRV concentrations in the aqueous phase after 90 min of dynamic in vitro lipolysis. These results demonstrated the importance of the more hydrophilic pore former PS65 for the modulation of release kinetics from lipid-coated drugs, as previously also investigated by Becker et al. (2016) [24].

As shown in Figure 5b, the digestion of blank FaSSIF resulted in a release of FFA that originating from the digestion of phospholipids present in the lipolysis medium (background measurement). The in vitro lipolysis of uncoated amorphous CRV particles resulted in a lower amount of FFA titrated than during the background measurement, possibly due to the neutralization of the FFA by the weak base CRV. When comparing the release of FFA from coated particles with the background measurement, no significant differences were observed until 15 min of digestion. After 30 min, an increased release of FFA from the tripalmitin coating containing 20% (*w/w*) PS65 was detected, with a delayed release of FFA from tripalmitin containing 10% (*w/w*) PS65. A decreased amount of hydrophilic pores not only reduced the contact area for the aqueous media to the drug core but also might have affected the accessibility of the pancreatic lipase to the tripalmitin, resulting in a delayed release of FFA for the 10% (*w/w*) PS65 coating. However, it can be suspected that the PS65 content has a greater impact on the drug release than the lipid digestion itself, as the maximum drug concentration for uncoated and coated amorphous particles (20% (*w/w*) PS65) was reached during the first 30 min of in vitro lipolysis, a time point where no significant lipid digestion was measured.

## 4. Conclusions

The current study demonstrated the feasibility of coating neat amorphous drug particles with lipid excipients in a fluid bed coater by a solvent-free hot melt coating process. A coherent coating layer of tripalmitin containing 10% (*w/w*) and 20% (*w/w*) of PS65 on the surface of neat amorphous CRV particles was achieved. Dynamic in vitro lipolysis studies demonstrated the ability of the pancreatic lipase to digest the coating layer and showed an immediate release profile from amorphous particles coated with a high PS65 concentration. The applied coating had a negative impact on the physical stability of the amorphous core in comparison to uncoated amorphous CRV particles during storage and resulted in the formation of the crystalline CRV hydrate when being exposed to high relative humidity, indicating a permeability for the moisture of the coating layer. SEM-EDX analysis suggested the recrystallization of amorphous CRV at the interface between the crystalline lipid coating layer and the amorphous core, with CRV crystals growing in an upward direction while retaining the amorphous character of the remaining CRV core. These results highlight the importance of surfaces mediated processes for the physical stability of the amorphous form. Further studies will investigate lipid/emulsifier recrystallization as a potential trigger for the recrystallization of amorphous drugs.

## Supplementary Materials

The following are available online at https://www.mdpi.com/1999-4923/12/6/519/s1, Figure S1: Schematic of the coating instrument, Figure S2: XRPD diffractograms of reference materials, Figure S3: Chemical structures of CRV, tripalmitin and PS65, Figure S4: DSC thermograms of reference materials, Table S1: Detected thermal events in reference materials.
