# Supplementary material for: Hot Melt Coating of Amorphous Carvedilol
Source: Pharmaceutics. 2020 Jun 6;12(6):519. doi: 10.3390/pharmaceutics12060519 (PMC7356097; doi:10.3390/pharmaceutics12060519)
Supplement: Supplementary file 1 [file pharmaceutics-12-00519-s001.pdf]

Supplementary Materials: Hot Melt Coating of Amorphous Carvedilol

Jacob Bannow, Lina Koren, Sharareh Salar-Behzadi, Korbinian Löbmann, Andreas Zimmer and Thomas Rades

1. Hot Melt Coating (HMC) Setup

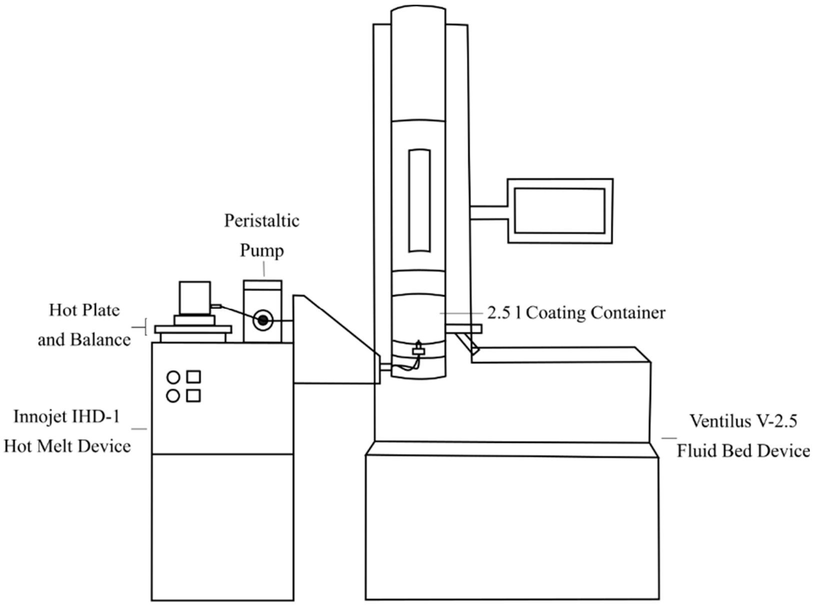

Figure S1. Schematic of the coating instrument.

2. Physical Stability Study

2.1. X-Ray Powder Diffraction (XRPD)

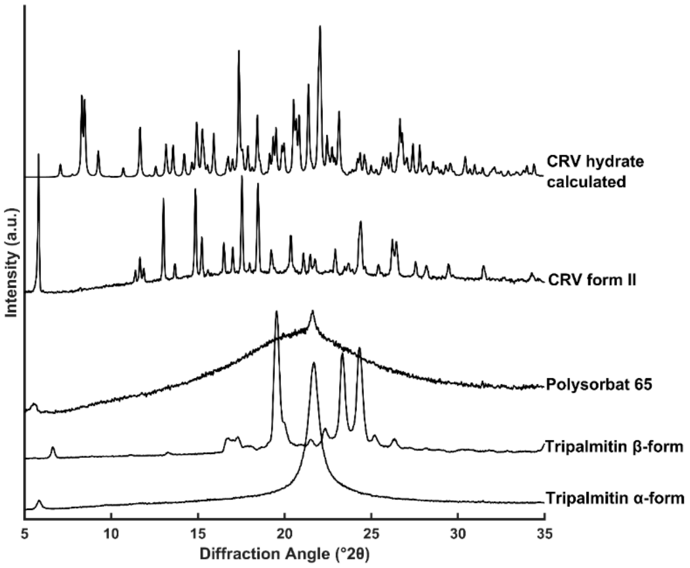

Figure S2. XRPD diffractograms of reference materials.

2.2. Chemical Composition of Cross-Sections

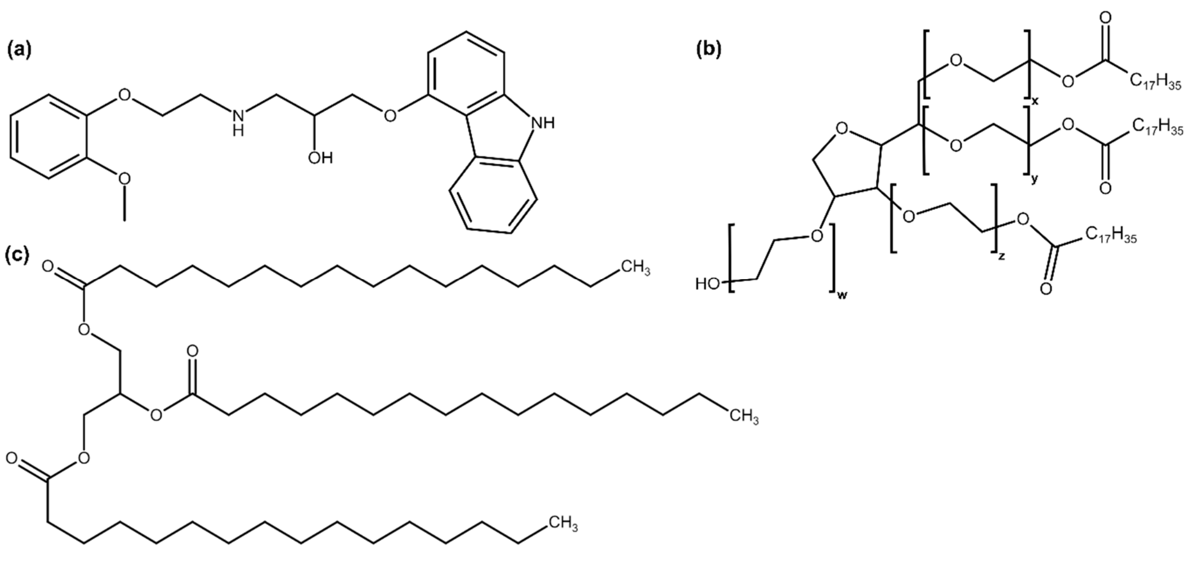

Figure S3. Chemical structures of (**a**) carvedilol, (**b**) tripalmitin and (**c**) PS65.

3. Differential Scanning Calorimetry (DSC)

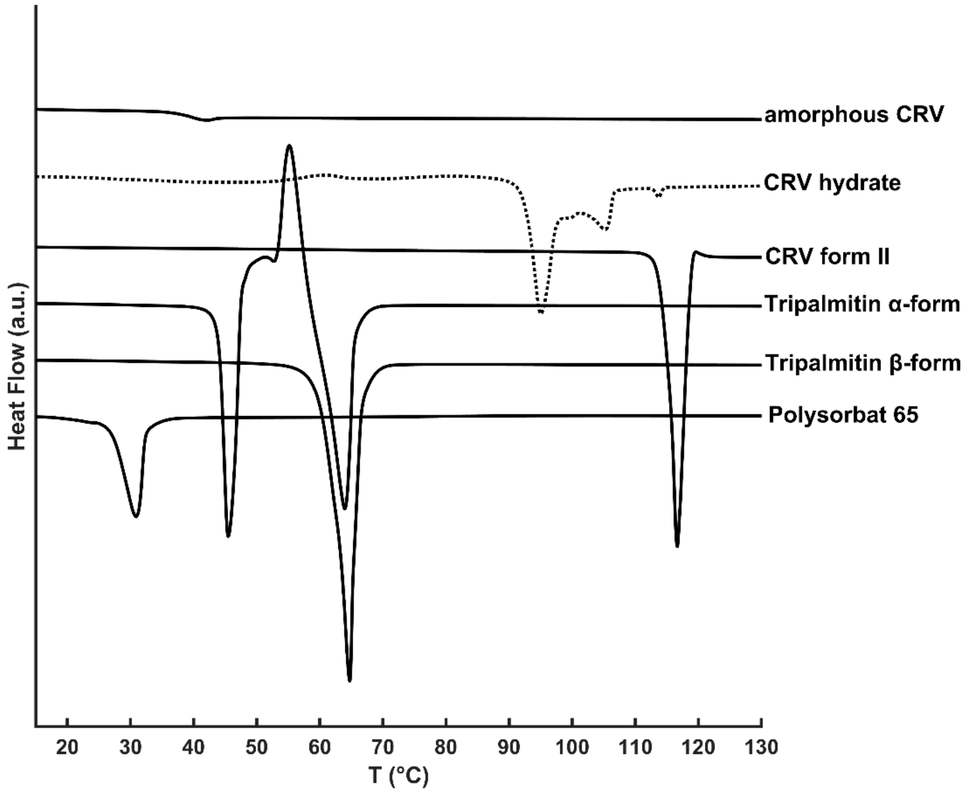

Figure S4. DSC thermograms of reference materials.

Table S1. Detected thermal events in reference materials.

Reference Materials	T_g (°C)	T_m onset (°C)	$T_m$peak (°C)
Polysorbat 65	-	26.9	30.9
Tripalmitin β	-	61.7	64.7
Tripalmitin α	-	44.0	45.5
Carvedilol (CRV) form II	38.6	114.9	116.7
CRV hydrate (not pure)	-	92.5	95.0
